# Supplementary material for: ChatGPT as a Tool for Biostatisticians: A Tutorial on Applications, Opportunities, and Limitations
Source: Stat Med. 2025 Oct 23;44(23-24):e70263. doi: 10.1002/sim.70263 (PMC12548020; doi:10.1002/sim.70263)
Supplement: Supplementary file 1 — Data S1. Supporting Information. [file SIM-44-0-s001.zip › Supplementary Materials Additional.zip!/3.5_Causal_Inferece/3.5_Causal_Chat9.pdf]

Combining statistical and causal thinking_9 - Biostatistics Expert Role

Please clear memory.

I've cleared the memory of our past interactions. If there's anything new you'd like to work on, feel free to let me know!

Take the role of an expert biostatistician for this Chat

Understood. I'll take the role of an expert biostatistician for this chat. Let me know what you'd like help with—whether it's study design, statistical analysis, causal inference, regulatory considerations, or something else.

I have data from a randomised clinical trial. I want to compare the efficacy of the experimental treatment with that of the control on overall survival in patients with non-small cell lung cancer. In this two-arm RCT, a considerable number of patients in the control arm switched to the experimental treatment after disease progression, while those in the experimental arm never switched.
My goal is to estimate the causal effect of sustained intake of the experimental treatment versus sustained intake of control on Overall survival, had the control group patients not been allowed to switch. Based on the information provided above, please define my target Estimand, following the guidance from the Estimand framework (ICH-E9 addendum)

To define your target estimand following the **ICH E9(R1)** Addendum on estimands and sensitivity analysis in clinical trials, we will clearly articulate each of the five attributes: **treatment, population, variable (endpoint), intercurrent events (with strategy), and summary measure**.

Given your setting—a two-arm randomised controlled trial (RCT) in non-small cell lung cancer (NSCLC) with treatment switching in the control arm post-progression—the appropriate estimand to reflect your *causal* research question is a **hypothetical strategy estimand**.

🔹 Target Estimand Definition

Treatment:

- *Sustained* treatment with the experimental therapy versus sustained treatment with the control therapy (as randomised), regardless of post-progression status.

Population:

- All randomised patients with NSCLC enrolled in the trial, consistent with the *intention-to-treat (ITT)* population.

Variable (endpoint):

- **Overall survival (OS)**: Time from randomisation to death from any cause.

Intercurrent Events (ICEs):

- **Treatment switching** in the control arm after disease progression.
  - *Strategy:* **Hypothetical** — you aim to estimate what OS would have been if **no patients had switched to the experimental treatment** after progression.
- **Other ICEs** (e.g. treatment discontinuation, non-cancer deaths, etc.) should also be specified. If your interest is only in switching, these can be addressed with treatment policy or appropriate strategies.

Summary Measure:

- Difference in survival distributions (e.g., **hazard ratio**, **restricted mean survival time**, or **difference in median OS**) between the two groups under the hypothetical no-switch scenario.

📌 Example (Structured Estimand Statement)

> In patients with NSCLC randomised to experimental or control treatment, we aim to estimate the causal effect of **sustained experimental treatment versus sustained control**, on **overall survival**, under a **hypothetical scenario** where **control patients had not switched to experimental treatment after disease progression**. The estimand targets a **contrast in overall survival distributions**, such as a hazard ratio or difference in restricted mean survival time.

Would you like help operationalising this estimand (e.g., identifying appropriate causal methods such as IPCW, g-formula, or RPSFTM) or aligning it with your statistical analysis plan?

The switching behaviour is affected by a set of different baseline and time-varying covariates. Here, I want to assume that the time-varying confounders that influence the switching behaviour and Overall survival are also themselves affected by prior treatment intake.
For e.g. ECOG score might not only affect whether patients should switch treatment or not, but its value might also be affected by prior treatment intake reflected by the switching behaviour.
In this case, the analytical problem becomes more complex. Can you explain?

You're absolutely right—and you've identified a **classic case of time-varying confounding affected by prior treatment**, which makes the analytical problem **non-identifiable via standard regression approaches**.

Let me break this down and explain why it becomes more complex, both conceptually and analytically.

🔹 What's Happening?

In your setting:

- Patients in the **control arm** may switch to the experimental treatment **after progression**.
- The **decision to switch** is influenced by **time-varying covariates** (e.g., ECOG performance status, tumour size, progression status).
- These **time-varying covariates** are themselves **affected by prior treatment exposure** (e.g., prior switching or staying on control).
- Both the time-varying covariates and the switching affect **Overall Survival (OS)**.

🔹 Why Is This a Problem?

This creates **treatment-confounder feedback**—a form of **time-dependent confounding** that is **affected by prior treatment**:

- At each time point, your decision to switch (or not) is influenced by variables like ECOG, which are **predictors of survival**.
- But ECOG itself is **influenced by prior treatment history** (e.g., receiving or not receiving experimental treatment).
- So if you simply condition on ECOG (e.g., via Cox regression or a time-dependent Cox model), you **block part of the treatment effect**—this is **adjustment bias**.

Result:
Standard regression approaches, including time-dependent Cox models, **cannot be used** to estimate the causal effect of sustained treatment strategies under such feedback loops. They will **not recover the target estimand** because they **control for mediators and collider paths** introduced by the feedback.

🔹 Why G-methods Are Needed

To properly estimate the **hypothetical estimand** of sustained experimental vs sustained control treatment, **causal inference methods that can handle time-varying confounding affected by prior treatment are required**.

These include:

1. **Inverse Probability of Treatment Weighting (IPTW)**
   - Weights are used to create a pseudo-population where treatment assignment (including switching decisions) is independent of measured confounders.
   - Requires correct modelling of the **treatment switching mechanism** and **censoring**.
2. **G-formula (Parametric G-computation)**
   - Models the entire longitudinal data structure (treatment, covariates, outcome).
   - More flexible but computationally intensive and sensitive to model misspecification.
3. **G-estimation of Structural Nested Models (e.g., SNFTMs or RPSFTMs)**

- Specifically designed to handle treatment switching.
- Makes assumptions about a common treatment effect ("treatment rank preservation") and generally requires **no unmeasured confounding**.

4. **Targeted Maximum Likelihood Estimation (TMLE)**
    - Doubly robust approach that can also be applied, although less common in switching contexts.

🔹 Conceptual Illustration (Simplified DAG)

```plaintext
       L0        →       L1         →        L2         →   OS
        ↓                 ↓                   ↓                ↑
  A0 (RANDOMISED)    A1 (Switch?)      A2 (Post-switch)    (feedback)
        →─────────────→─────────────→─────────────→─────────────→
              ↑             ↑              ↑
              └─────────────┴──────────────┘
        (Time-varying confounders affected by prior A)
```

Where:

- `L1`, `L2` = time-varying covariates (e.g. ECOG)
- `A1`, `A2` = treatment received (0 = control, 1 = experimental)
- `OS` = overall survival
- The arrows from `A` to `L` and `L` to `A` show **treatment-confounder feedback**.

🔹 Summary

Your setting requires **methods that respect the temporal structure** and **account for time-varying confounding affected by prior treatment**. Naive adjustment **breaks causal pathways** and leads to biased estimates.

Let me know if you'd like to discuss:

- How to specify models for IPTW or g-formula in your setting;
- Which assumptions are needed for identification and estimation;
- A simulation or causal diagram illustrating your case;
- How to practically implement these methods in R (e.g., `ipw`, `ltmle`, `gfoRmula` packages).

Depict our research problem in a DAG

Based on your setting—an RCT in non-small cell lung cancer (NSCLC) with treatment switching in the control arm, influenced by **baseline and time-varying covariates**, where those time-varying covariates are also affected by prior treatment—we can draw a **causal DAG** that illustrates:

- Baseline confounding (`L0`)
- Time-varying confounding (`L1`, `L2`)
- Treatment switching (`A1`, `A2`)
- Outcome (`Y`, overall survival)
- Feedback loops from prior treatment to covariates
- Confounding of both treatment and outcome by the covariates

---

🔹 DAG (Conceptual Representation)

```plaintext
         L0
        /  \
       ↓    ↓
      A0 →  L1 → A1 → L2 → A2 → Y
            ↑     ↑     ↑     ↑
            └─────┴─────┴─────┘
```

🔹 Variable Definitions

- `L0`: Baseline covariates (e.g. age, baseline ECOG, CNS involvement)
- `A0`: Initial randomised treatment (0 = control, 1 = experimental)
- `L1`, `L2`: Time-varying covariates (e.g. updated ECOG, progression status, tumour size)
- `A1`, `A2`: Treatment taken at time 1 and 2 (may reflect switching from control to experimental)
- `Y`: Overall survival (OS), which is affected by cumulative treatment and time-varying factors

---

🔹 Key Features

- **Randomisation (`A0`)** breaks confounding at baseline.
- However, **switching decisions (`A1`, `A2`)** are **not random** and are influenced by time-varying covariates (`L1`, `L2`).
- These covariates (`L1`, `L2`) are **affected by prior treatment** (`A0`, `A1`), creating **treatment-confounder feedback**.
- Standard regression adjusting for `L1`, `L2` will **block part of the treatment effect** (conditioning on post-treatment variables that lie on the causal pathway).

🔹 Implications

This DAG shows that:

- **Naive adjustment for time-varying confounders** (e.g. ECOG or tumour size) introduces bias.
- We require **G-methods** to adjust correctly for the feedback loop between treatment and confounding.

- Estimating the **causal effect of sustained treatment** (i.e. "had all control patients remained on control") requires carefully modelling or weighting these pathways.

If you like, I can generate a **graphical DAG image** of this structure. Would you like me to do that?

I would like to apply the inverse probability of censoring weighting (IPCW) approach to address the bias introduced by switching following the methodology discussed in the following paper: Robins JM, Finkelstein DM. Correcting for noncompliance and dependent censoring in an AIDS clinical trial with inverse probability of censoring weighted (IPCW) log-rank tests. Biometrics. 2000;56(3):779–88.
Briefly summarise the key points from this paper

Certainly. The paper by **Robins and Finkelstein (2000)** is foundational for the **Inverse Probability of Censoring Weighting (IPCW)** approach. Here's a concise summary of the key points, structured to connect directly with your interest in handling treatment switching in clinical trials:

🔹 Context and Objective

- The authors address **noncompliance** (i.e. deviation from assigned treatment) and **dependent (informative) censoring** in RCTs.
- Standard methods (e.g. ITT, per-protocol) can yield biased estimates when **treatment received** is influenced by **time-varying covariates** that also affect the outcome.
- Their motivating example is an AIDS clinical trial, but the methodology is applicable to any setting with **time-varying treatment and confounding**, including your NSCLC trial with **treatment switching**.

🔹 Core Idea of IPCW

- The authors propose to **censor patients at the time they deviate from the target treatment strategy**, e.g., switch treatments.
- Since this **artificial censoring** is **not independent of prognosis**, naive survival analyses on the censored data are biased.
- To correct for this, they introduce **IPCW**, which reweights the remaining uncensored patients to represent those who would have remained uncensored **under a hypothetical regime** (e.g., no switching).

🔹 How IPCW Works

1. **Define a hypothetical strategy** (e.g., always remain on randomised treatment).
2. **Artificially censor** patients at the time they **deviate** (e.g., switch treatment).
3. Estimate **censoring probabilities** using observed covariates (baseline + time-varying).

- These are estimated using a **pooled logistic regression** or similar model.

4. Compute **inverse probability weights**:

$$w_i(t) = \frac{1}{\hat{P}(C_i > t \mid \text{history up to } t)}$$

   where C_i is the artificial censoring time.

5. Use these weights in a **weighted log-rank test** or **weighted Cox model** to estimate the causal effect.

🔹 Assumptions for IPCW to be Valid

1. **No unmeasured confounding**: All variables that influence both censoring (i.e. switching) and the outcome are observed and correctly modelled.
2. **Positivity**: Every patient has a nonzero probability of remaining uncensored at each time point.
3. **Correct model specification**: The censoring model must be correctly specified to estimate the weights accurately.
4. **Consistency**: The observed data accurately represent what would happen under the hypothetical scenario (e.g., perfect adherence to control).

🔹 Advantages

- Allows valid estimation of causal effects under **hypothetical treatment strategies**.
- Accommodates **time-varying confounding affected by prior treatment**, unlike standard Cox models.

🔹 Limitations

- Sensitive to **model misspecification** of the censoring model.
- Weights can become **unstable or extreme**, especially with rare switching or positivity violations.
- Doesn’t provide insight into mechanisms of treatment effect; it's a marginal method (not conditional).

🔹 Relevance to Your Study

This IPCW framework allows you to **estimate the causal effect of remaining on randomised treatment**, even when many control patients **switch** after progression, as long as switching is **adequately modelled** using baseline and time-varying covariates.

Would you like help constructing the weights in R or specifying the censoring model based on your available covariates?

Could you elaborate more on the research question Robins and Finkelstein were trying to answer in their paper, mentioning the comparator arms, possible intercurrent events, outcome definition and population under study.

Certainly. Let's walk through the **research question** that Robins and Finkelstein addressed in their seminal 2000 paper, using the **estimand framework-style structure** (even though that terminology came later). This structure helps align their objectives with your context.

🔹 Study Context and Objective

Robins and Finkelstein aimed to estimate the **causal effect** of **Zidovudine (ZDV) vs. placebo** on **AIDS-free survival** in patients enrolled in a clinical trial, while accounting for **noncompliance** and **informative censoring** due to treatment switching and loss to follow-up.

🔹 1. Treatment

- **Experimental arm:** Zidovudine (ZDV), an antiretroviral drug.
- **Control arm:** Placebo.

Patients were **randomised** to one of these two treatment arms, but could later **deviate** from the assigned treatment—for example, placebo patients could begin ZDV outside the trial protocol (e.g., due to disease progression or physician discretion).

🔹 2. Population

- HIV-positive individuals enrolled in an AIDS clinical trial.
- The full **intention-to-treat (ITT)** population was considered, but the **estimand** of interest reflects a **per-protocol-like**, causal contrast under **full adherence** to randomised treatment.

🔹 3. Variable (Endpoint)

- **Outcome:** Time to AIDS diagnosis or death, referred to as **AIDS-free survival**.
- This is a **time-to-event** variable, measured from randomisation.

🔹 4. Intercurrent Events

The authors focus on two types of **intercurrent events** that could bias naive analyses:

1. **Treatment switching / noncompliance:**
    - Patients assigned to placebo may **initiate ZDV** during follow-up.
    - This switching is **not random**; it's often driven by **worsening health**, which is also predictive of the outcome.
2. **Loss to follow-up / dropout:**

- Patients may drop out of the study for reasons related to their health status or treatment experiences.
- This leads to **informative censoring**, as the probability of dropout is **not independent** of the event time.

---

5. Estimand (Implicit)

The implicit estimand aligns with a **hypothetical strategy estimand** under modern terminology:

> *"What would be the difference in AIDS-free survival between patients if all adhered to their assigned treatment throughout follow-up, and there was no switching or dropout?"*

This causal question requires adjusting for **noncompliance** and **dependent censoring** using appropriate methods.

Analytical Challenge

Treatment switching and dropout are **dependent** on **time-varying covariates** (e.g., CD4 count), which are themselves **affected by prior treatment**. Therefore, standard methods (like Cox models with covariate adjustment) are **invalid** due to:

- **Time-varying confounding affected by prior treatment**
- **Informative censoring**

Solution Proposed

The authors propose an **IPCW log-rank test**, which involves:

- Artificially censoring patients at the time of **treatment switching or dropout**
- Estimating **inverse probability weights** based on observed covariates to correct for informative censoring
- Using these weights in a **weighted survival analysis** to recover the **causal effect of assigned treatment**

Summary of Their Research Question

> **In HIV-positive patients randomised to ZDV or placebo, what is the causal effect of remaining on the assigned treatment throughout follow-up on AIDS-free survival, in the presence of noncompliance and dropout influenced by health status?**

This parallels your interest in estimating the causal effect of **sustained treatment with experimental therapy vs control** on **overall survival**, under a **hypothetical no-switching scenario**.

Let me know if you'd like a side-by-side mapping of their setup to yours or help specifying the IPCW model in your data.

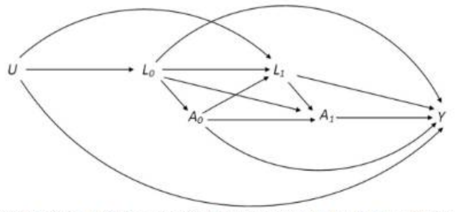

Fig. 1 Graphical representations of our research focus and the bias of naive methods for time-varying confounding in the presence of treatment-confounder feedback: This Directed acyclic graph (DAG) represents the post-progression period for patients in the control arm. Following disease progression, the decision as to whether or not a patient in the control group continues to take its treatment (A_0) or (A_1) depends on predictive factors measured at or before the time of disease progression (L_0). Again, we assume that treatment intake (A_0) affects outcome (Y) and influences a set of TVCs at a later time-point (L_1). These covariates are also assumed to impact the clinician's decision as to whether or not a patient should subsequently change treatments (A_1) introducing time-varying confounding and treatment-confounder feedback through L_1

Attached, I provide you with a directed acyclic graph (DAG) that represents our research problem.
To simplify this DAG and take into consideration this deterministic information provided below, I restrict my DAG to the post-progression period for patients in the control arm only and consider two follow-up time points, 1 & 2.
Here A(k) is deterministically 0 if A(k-1)=0 AND L(k-1) (reflecting progression status)=0 (no progression).
Following disease progression, the decision as to whether or not a patient in the control group continues to take their treatment (A0 or A1) depends on predictive factors measured at or before the time of disease progression (L0). These covariates are also assumed to impact the clinician's decision as to whether or not a patient
should subsequently change treatments (A1), introducing time-varying confounding and treatment confounder feedback through L1.
I will emphasise again that in our case scenario, patients in the control group do not switch unless they progress, and all patients in the experimental group never switch.
I still want to estimate in my outcome model the causal effects of sustained treatment interventions (always taking control versus always taking experimental) under a hypothetical strategy where control patients do not switch treatment.
I want you to use this DAG as a basis for performing the appropriate IPCW analyses and provide me with the analysis R-script.
You may assume that the dataset is in long format, with variables: id, time (in months and increases with increments of 1), death indicator, switching indicator, baseline covariates
repeated over time points, time-varying covariates at each time pt.

Again, we assume that treatment intake (A0) affects outcome (Y) and influences a set of TVCS at a later time-point (L1).

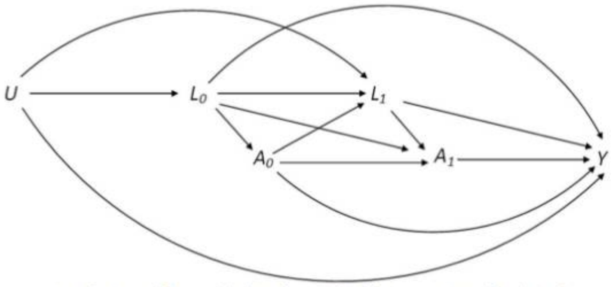

Fig. 1 Graphical representations of our research focus and the bias of naive methods for time-varying confounding in the presence of treatment-confounder feedback: This Directed acyclic graph (DAG) represents the post-progression period for patients in the control arm. Following disease progression, the decision as to whether or not a patient in the control group continues to take its treatment (A_0) or (A_1) depends on predictive factors measured at or before the time of disease progression (L_0). Again, we assume that treatment intake (A_0) affects outcome (Y) and influences a set of TVCs at a later time-point (L_1). These covariates are also assumed to impact the clinician's decision as to whether or not a patient should subsequently change treatments (A_1) introducing time-varying confounding and treatment-confounder feedback through L_1

Based on the DAG you've provided and the assumptions you've outlined (control patients only switch after progression, treatment affects future covariates, and time-varying confounding is present), the appropriate IPCW analysis censors patients at the time of switching and then reweights the uncensored patients using inverse probability of censoring weights to estimate the causal effect of sustained treatment strategies.

Below is a **template R script** for performing an **IPCW-weighted Cox proportional hazards model** using a dataset in **long format**.

🔹 R Script for IPCW Analysis

```r
# Required packages
library(survival)
library(ipw)
library(dplyr)

# Assume 'data_long' is your long-format dataset
# Variables: id, time, death, switch, A, L1, L0, and baseline covariates

# Step 1: Define censoring indicator (Censor at switching)
data_long <- data_long %>%
  group_by(id) %>%
  mutate(
    switch_time = ifelse(switch == 1, time, NA),
    switch_time = min(switch_time, na.rm = TRUE),
    switch_time = ifelse(is.infinite(switch_time), NA, switch_time),
    censored = ifelse(!is.na(switch_time) & time >= switch_time, 1, 0)
  ) %>%
  ungroup()

# Artificially censor at switching time
data_long <- data_long %>%
  filter(is.na(switch_time) | time < switch_time)

# Step 2: Fit model for censoring (i.e., switching) to compute IPC weights
# Predict probability of *not* being censored at each time
# Use logistic regression with covariate history (L0, L1, A, time, etc.)

censor_model <- glm(
  censored ~ L0 + L1 + A + time,
  data = data_long,
  family = binomial()
)

# Step 3: Predict probabilities and compute weights
data_long <- data_long %>%
  mutate(
    p_uncensored = 1 - predict(censor_model, type = "response"),
    ipcw_weight = 1 / p_uncensored
  )

# Step 4: Collapse data to individual-level for Cox model with IPC weights
# Take only the last time point per individual (i.e., time of death or censoring)
data_ipcw <- data_long %>%
  group_by(id) %>%
  filter(time == max(time)) %>%
  ungroup()

# Step 5: Cox model with IPC weights
cox_ipcw <- coxph(
  Surv(time, death) ~ A,
  data = data_ipcw,
  weights = ipcw_weight
)
```

```
summary(cox_ipcw)
```

🔹 Notes

- `A`: treatment received at baseline (0 = control, 1 = experimental).
- `switch`: indicator for treatment switch (1 = switched).
- `death`: indicator for death event (1 = death, 0 = alive).
- `L0`, `L1`: baseline and time-varying confounders (e.g. ECOG, tumour burden, etc.).
- This code **artificially censors** control-arm patients at switching and assumes experimental-arm patients never switch.
- This script can be extended to **multiple time points** and include **stabilised weights** or **more complex censoring models** if needed.

Would you like me to adapt this template further to your specific variables or provide diagnostics (e.g., weight distribution, positivity checks)?
